# Selective, high-contrast detection of syngeneic glioblastoma *in vivo*

**DOI:** 10.1038/s41598-020-67036-z

**Published:** 2020-06-19

**Authors:** Richard B. Banati, Paul Wilcox, Ran Xu, Grace Yin, Emily Si, Eric Taeyoung Son, Mauricio Shimizu, R. M. Damian Holsinger, Arvind Parmar, David Zahra, Andrew Arthur, Ryan J. Middleton, Guo-Jun Liu, Arnaud Charil, Manuel B. Graeber

**Affiliations:** 1 Australian Nuclear Science and Technology Organization, Locked Bag 2001, Kirrawee DC, NSW 2232 Australia; 2 Medical Imaging, Faculty of Medicine and Health, The University of Sydney, 94 Mallett Street, Camperdown, NSW 2050 Australia; 3 Brain Tumour Research, Brain and Mind Centre, Faculty of Medicine and Health, The University of Sydney, 94 Mallett Street, Camperdown, NSW 2050 Australia; 4 Molecular Neuroscience, Brain and Mind Centre, Faculty of Medicine and Health, The University of Sydney, 94 Mallett Street, Camperdown, NSW 2050 Australia

**Keywords:** Biological techniques, Cancer, Drug discovery, Neuroscience, Oncology

## Abstract

Glioblastoma is a highly malignant, largely therapy-resistant brain tumour. Deep infiltration of brain tissue by neoplastic cells represents the key problem of diffuse glioma. Much current research focuses on the molecular makeup of the visible tumour mass rather than the cellular interactions in the surrounding brain tissue infiltrated by the invasive glioma cells that cause the tumour’s ultimately lethal outcome. Diagnostic neuroimaging that enables the direct *in vivo* observation of the tumour infiltration zone and the local host tissue responses at a preclinical stage are important for the development of more effective glioma treatments. Here, we report an animal model that allows high-contrast imaging of wild-type glioma cells by positron emission tomography (PET) using [18 F]PBR111, a selective radioligand for the mitochondrial 18 kDa Translocator Protein (TSPO), in the Tspo^−/−^ mouse strain (C57BL/6-Tspo^tm1GuMu(GuwiyangWurra)^). The high selectivity of [18 F]PBR111 for the TSPO combined with the exclusive expression of TSPO in glioma cells infiltrating into null-background host tissue free of any TSPO expression, makes it possible, for the first time, to unequivocally and with uniquely high biological contrast identify peri-tumoral glioma cell invasion at preclinical stages *in vivo*. Comparison of the *in vivo* imaging signal from wild-type glioma cells in a null background with the signal in a wild-type host tissue, where the tumour induces the expected TSPO expression in the host’s glial cells, illustrates the substantial extent of the peritumoral host response to the growing tumour. The syngeneic tumour (TSPO^+/+^) in null background (TSPO^−/−^) model is thus well suited to study the interaction of the tumour front with the peri-tumoral tissue, and the experimental evaluation of new therapeutic approaches targeting the invasive behaviour of glioblastoma.

## Introduction

Microglia cells, the brain’s immune defence cells, populate gliomas in large numbers and are especially numerous in glioblastoma^1^. Microglia are so numerous in these neoplasms as to significantly contribute to the actual tumour mass^2^. While the occurrence of microglial cells in glioma has been known since 1925^3^, only recently have microglia attracted attention as a potential new treatment target^1,4^. One reason for this delay was a long-standing controversy about the identity of microglia, including their very existence^5^ that lasted until the 1990s when microglia became finally accepted as the brain’s resident immune cells^6^. Importantly, microglia have since also become recognized as an exquisitely sensitive “sensor of pathology”^7^. This property now forms the basis for the appreciation of activated microglia as a generic marker of active brain tissue changes, ranging from plasticity-related tissue remodelling^8,9^ to outright pathology in PET imaging studies^10,11^.

The central problem of malignant glioma including glioblastoma, the most common and lethal primary brain tumour, is the diffuse infiltration of brain tissue by the neoplastic glial cells^12^. After decades of research, unlike for other malignant cancers, such as melanoma^13^, survival statistics are still dire^12^.

The critical process of diffuse infiltration in particular remains poorly understood and has eluded direct *in vivo* observation, both in clinical cases and in preclinical models due to the difficulty in confidently distinguishing tumour tissue from the reactive normal host tissue within the infiltration zone^14–18^.

*In vivo* imaging by positron emission tomography (PET) of the mitochondrial 18 kDa Translocator Protein (TSPO), formerly known as the peripheral benzodiazepine binding site or receptor (PBR)^19,20^, has long been known to be highly expressed in tumour cells of glioblastoma^21–24^. However, microglia and, to a lesser extent, reactive astrocytes, too, are an important source of disease-induced TSPO expression in the pathologically altered microenvironment around a malignant glioma^25^. It is thus not readily possible to discern the relative contributions of infiltrating tumour cells from that of reactive glial cells to the total TSPO expression as measured by *in vivo* TSPO imaging.

This important experimental limitation, the inability to selectively detect and visualise *in vivo* glioma growing in the infiltration zone, can be overcome through the creation of a complete Tspo/PBR knockout mouse^11^ and the implantation of a TSPO expressing syngeneic infiltrative glioma.

## Materials and Methods

### Animals

The Tspo−/− mouse strain, C57BL/6-Tspo^tm1GuMu(GuwiyangWurra)^ has been described previously^11^. In order to produce genetic background-matched normal control animals for experiments, heterozygous mice were crossed to generate wild-type littermate controls. All animal procedures were approved by the University of Sydney Animal Ethics Committee and the ANSTO Animal Care and Ethics Committee. All methods were carried out in accordance with relevant guidelines and regulations.

### Implantation of GL261 cells

The GL261 mouse glioma cell line was obtained from the National Cancer Institute Tumour Repository, Frederick, MD, USA. For stereotactic implantation of the cells, the Tspo+/+ (n = 4) and Tspo−/− mice (n = 12) were operated under isoflurane anaesthesia using a Model 900 Small Animal Stereotaxic Instrument and a Model 1911 Stereotaxic Drill (David Kopf Instruments, Tujunga, CA, USA). A small burr hole was made 3 mm anterior to the bregma and 2 mm lateral over the right hemisphere. GL261 glioblastoma cells were injected slowly to a depth of 3 mm ventral at 3 ×10^3^ per µl in a volume of 5 µl over 2 min using a 10-µl Hamilton syringe connected to a pump by means of a 32-gauge needle. The animals were monitored daily for good recovery and absence of neurological signs. Three and four weeks after the operation, the animals were imaged by means of microPET/CT and the brains were dissected for histological studies and autoradiography.

### PET/CT and MR imaging

Mice, anaesthetized (5% (v/v) isoflurane and maintained at 1–2%), were scanned using a small-animal Inveon PET/CT scanner (Siemens, Knoxville, TN) following methods described previously^26,27^. Body temperature was maintained with a feedback regulated heating pad and respiration monitored (BioVet; m2m Imaging Corp, Cleveland, OH). Scans started with a tail vein injection of [18 F]PBR111 (8–18 MBq/100 μL, 0.2 nM) and after 40 minutes of imaging data acquisition concluded with a 10-min CT scan for anatomical co-registration information. All PET data were corrected, normalized and reconstructed with an OSEM3D–MAP algorithm^11^ to generate PET volumes of activity concentration (kBq/ml). The brains were removed after the PET scan and fixed in 4% formaldehyde in phosphate-buffered saline for 5 days prior to a sample being scanned by MRI.

MR imaging was performed at the University of New South Wales’ Biological Resources Imaging Laboratory (BRIL) at room temperature (22 °C) on a 9.4 T Bruker (Germany) BioSpec Avance III 94/20 preclinical magnetic resonance microimaging system equipped with a 15-mm radiofrequency coil. Sample chamber temperature was within the range 20.7–21.0 °C throughout the imaging process. The brains were scanned in coronal orientation using a 3D multi gradient-echo (MGE) sequence wherein the read gradient was oscillated to generate a series of eight echoes with the following echo times TE = {3.0604, 7.0604, 11.0604, 15.0604, 19.0604, 23.0604, 27.0604, 31.0604 msec}. Other parameters: TR = 80 msec, FOV = 15 × 15 × 8 mm, matrix size: 150 × 150 × 80 resulting in an isotropic resolution of 100 × 100 × 100 μm, FA = 30 deg, 8 acquisitions, measurement time = 2:08 h per sample). The PET/CT and MR image volumes were co-registered using Siemens Inveon Research Workplace (IRW, version 4.2; https://inveon-research-workplace.software.informer.com/4.0/). Only rigid scaling, rotation, and translation was applied for co-registration between the MR and PET/CT images.

### Histology and Immunohistochemistry

Paraffin sections were cut at 4 μm thickness and stained for hematoxylin and eosin: xylene for 2 minutes twice, 100% ethanol for 2 minutes, 95% ethanol for 2 minutes followed by a rinse in distilled water, haematoxylin for 4 minutes twice followed by a rinse with distilled water, acid alcohol (70% ethanol, 1% HCl) for 30 seconds followed by a rinse with distilled water, Leica Surgipath Blue Buffer 8 for 1 min, followed by a rinse with distilled water, eosin for 3 minutes, 95% ethanol for 2 minutes, 100% ethanol for 2 minutes, xylene for 2 minutes twice followed by mounting in DPX and cover-slipping.

For immunohistochemistry, sections were deparaffinized and rehydrated as described above but 90% ethanol was used for 2 minutes after the 100% ethanol step, followed by 70% ethanol for 2 minutes, 0.01 M PBS, pH 7.4, for 5 minutes twice, blocking with normal serum for 20 min, and incubation with the primary antibody (anti-GFAP or anti-Iba1) in a humid chamber overnight at 4 °C. Subsequently, sections were gently washed in 0.01 M PBS, pH 7.4, for 5 minutes twice, incubated with the secondary biotinylated antibody in a humid chamber at room temperature for 45 minutes followed by gentle washes in 0.01 M PBS, pH 7.4, for 5 minutes twice and incubation with ABC reagent in a humid chamber at room temperature for 45 minutes. This was followed by gentle washes in 0.01 M PBS, pH 7.4, for 5 minutes twice and 0.01 M TBS, pH 8.0, for 5 minutes, respectively. Detection of ABC-peroxidase complex to visualize antibody binding was performed using DAB solution followed by a rinse with water. Sections were briefly counterstained with hemalum, dehydrated, mounted in DPX and cover-slipped. Antibody dilutions used were 1:100 for monoclonal anti-GFAP (glial fibrillary acidic protein; mouse monoclonal, Sigma–Aldrich, St. Louis, Cat No. G 3893) or 1:500 for rabbit polyclonal anti-Iba1 (Wako, Osaka, Japan). VECTASTAIN ABC-Peroxidase Kits 4001 and 4002, respectively, were from Vector Laboratories, Burlingame, CA 94010, USA.

## Results

### [18 F]PBR111 PET/CT

**Figure 1:**
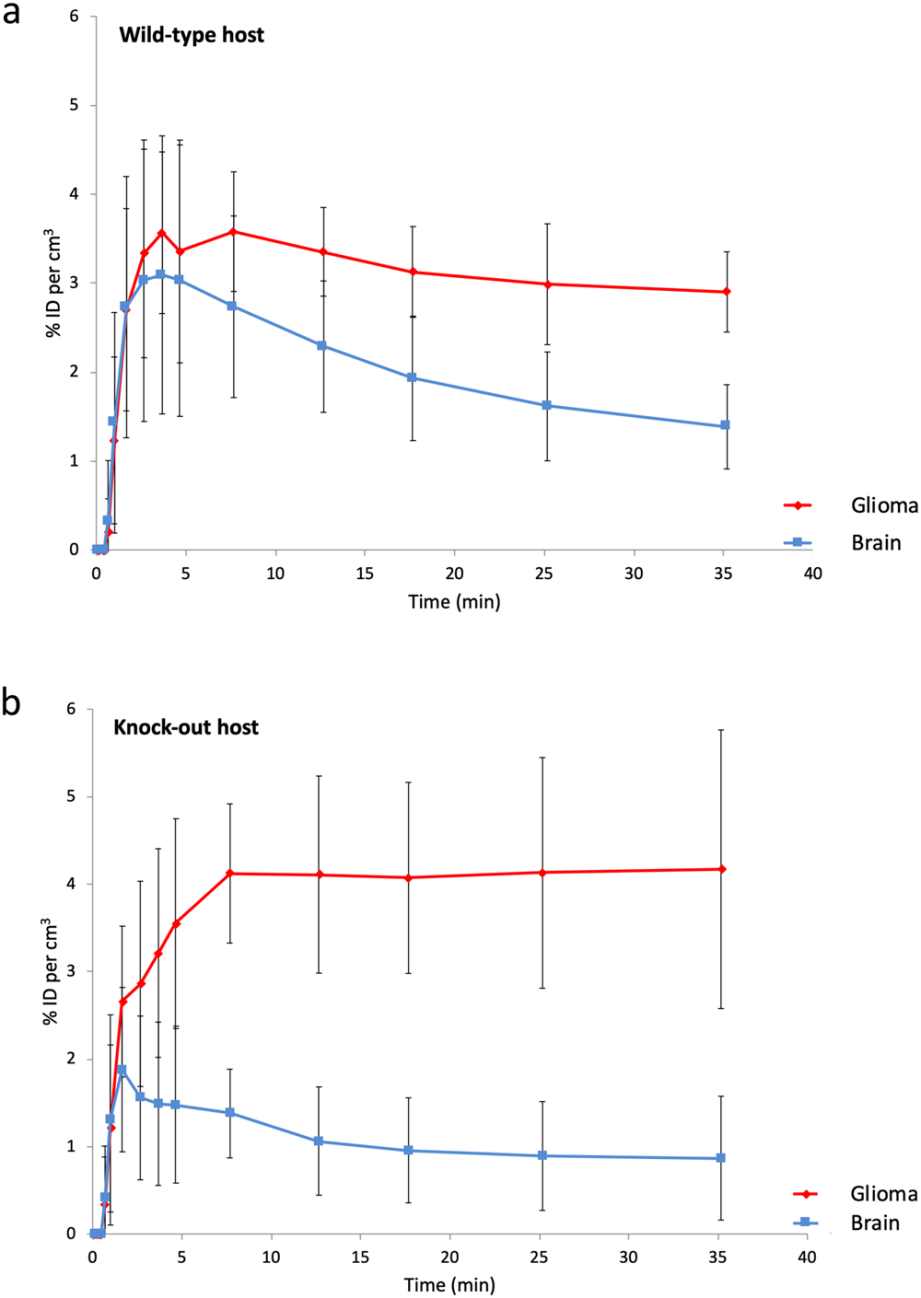
Tracer kinetic curves showing the behaviour of the ligand PBR111 in implanted TSPO expressing glioma (red), compared to the ligand kinetics of PBR111 in the tissue of the surrounding brain (blue). (**a**) Wild-type host: The kinetics of [18 F]PBR111 demonstrate the increased retention of [18 F]PBR111 in the TSPO expressing syngeneic tumour (red), while healthy, normal cerebral tissue (blue) (excluding cerebellum) in Tspo+/+ animals has low to absent [18 F]PBR111 retention. ID = injected dose; n = 3; error bars denote standard deviation. (**b)** Knock-out host: Tspo−/− mice do not have any specific binding of [18 F]PBR111 in any organ. ID = injected dose; n = 3; error bars denote standard deviation.

**Figure 2:**
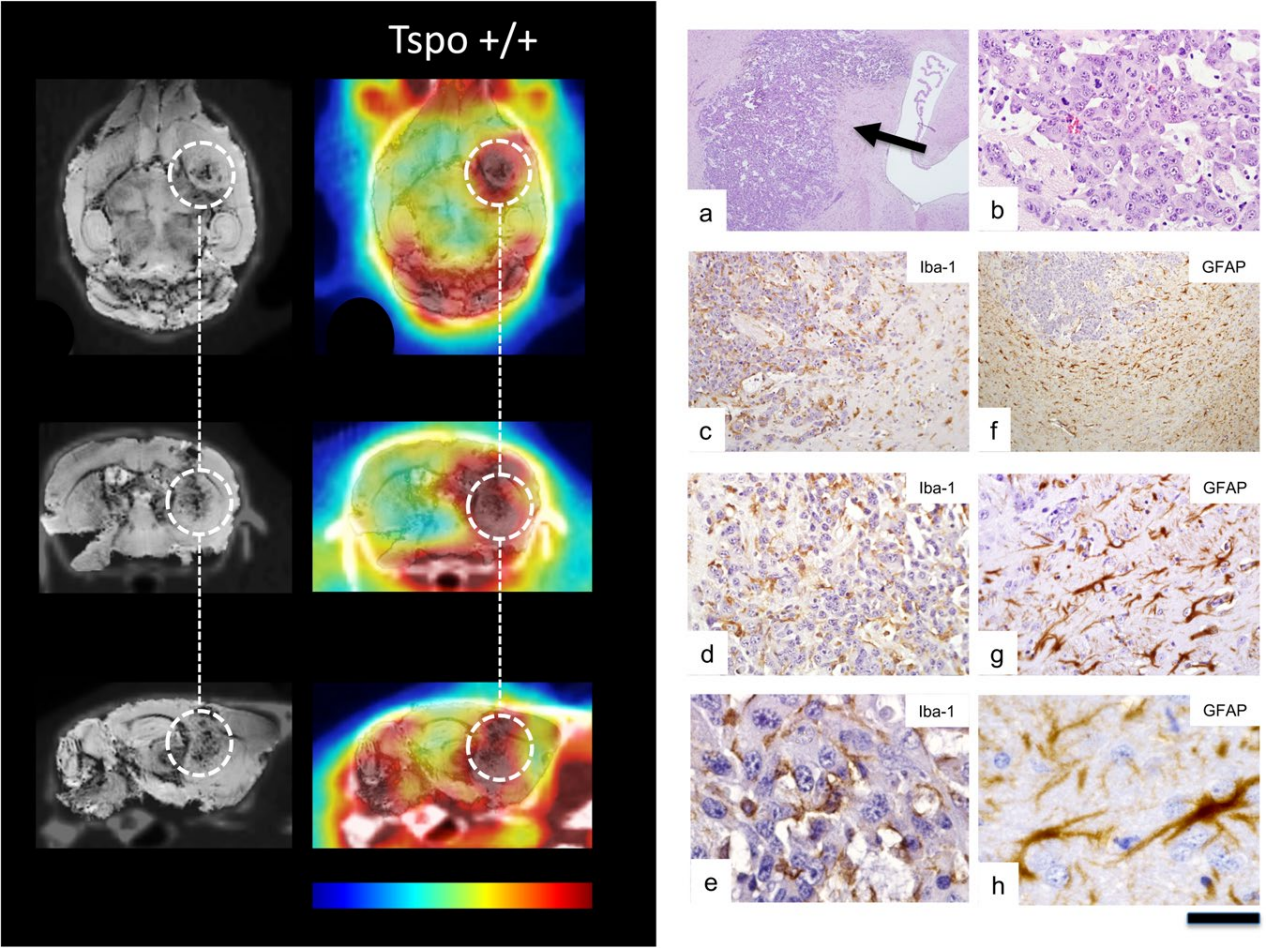
MicroPET/MRI/CT using TSPO ligand PBR111 in a Tspo+/+ mouse followed by histological examination. Imaging panel on the left-handed side. Left column: MR images (echo time 31.0604 msec) co-registered to CT images. Right column: PBR111-PET co-registered to MR-CT. The time-point frame of the PET images is 20–30 min after injection of ligand and scaling is 0–4% ID per cm^3^. The encircled area marks the location of the GL261 glioblastoma. Colour bar: The images are displayed with the same colour scaling and are directly comparable (highest PET values are red). Histology panel on the right. (**a)** Routine H&E staining shows GL261 glioma cells diffusely infiltrating brain tissue (arrow). (**b)** Many mitoses can be seen at high magnification. (**c–e**) Iba1 immunostaining reveals numerous microglia/macrophages within and around the experimental glioma. Notably, immunoreactive microglia/macrophages closely surround and even wrap tumour cells in the wild-type host indicating an intimate relationship between microglia/macrophages and glioma cells. (**f–h**) Labelling for the glial fibrillary acidic protein (GFAP) shows a strong astrocyte response around the tumour. Scale bar: 1 mm in (**a**), 100 µm in (**b**), 250 µm in (**c**), 500 µm in (**f**), 100 µm in (**d**,**g**), and 25 µm in (**e**,**h**).

**Figure 3:**
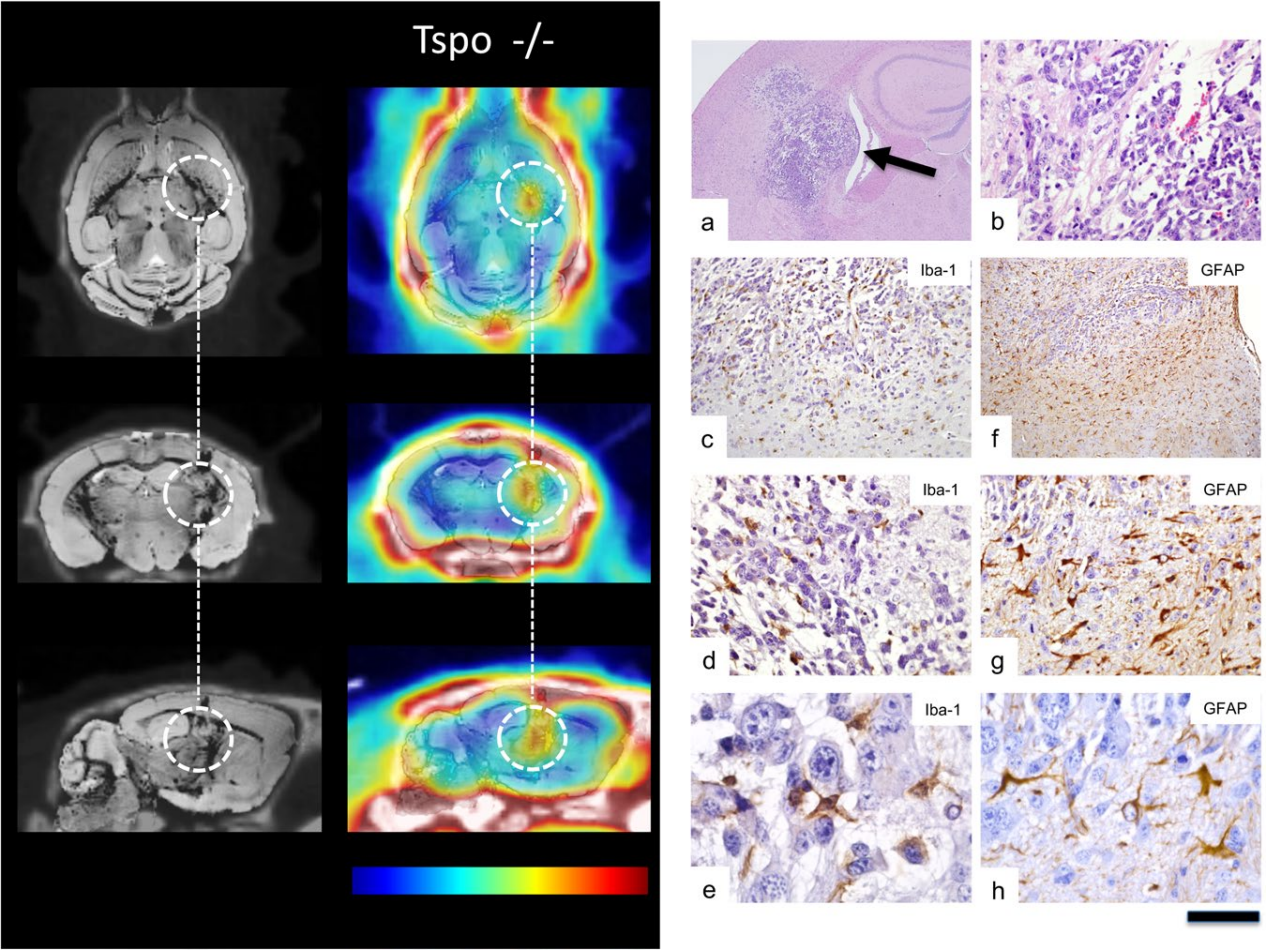
MicroPET/MRI/CT using TSPO ligand PBR111 in a Tspo knock-out mouse (Tspo−/−) followed by histological examination. Imaging panel on the left-handed side. Left column: MR images (echo time 31.0604 msec) co-registered to CT images. Right column: PBR111-PET co-registered to MR-CT. The time-point frame of the PET images is 20–30 min after injection and scaling is 0–4% ID per cm^3^. The encircled area marks the location of the GL261 glioblastoma. Since the cells in the tissue surrounding the TSPO-expressing tumour are not capable of expressing TSPO, the image solely delineates tumour tissue and unlike in Tspo^+/+^ animals (Fig. 2) there is no contributory signal from other cells, in particular not from activated microglia. Colour bar: The images are displayed with the same colour scaling and are directly comparable (highest PET values are red). Histology panel on the right. (**a**) In the TSPO knock-out animal, H&E staining also shows GL261 glioma cells diffusely infiltrating brain tissue (arrow), but tumour cells appear less densely packed than in the wild-type host. (**b**) Many mitoses can be seen. (**c–e**) Iba1 immunostaining reveals microglia/macrophages within and around the experimental glioma, but many are rounded, i.e. have fewer cell processes than in the wild-type host (cf. Fig. 2) and only few are in close contact with tumour cells. (**f–h**) The GFAP response is also weaker than in the wild-type host and a number of astrocytes look dystrophic. Scale bar: 1 mm in (**a**), 100 µm in (**b**), 250 µm in (**c**), 500 µm in (**f**), 100 µm in (**d**,**g**), and 25 µm in (**e**,**h**).

Tracer kinetic analysis (Fig. 1a,b) and image inspection (Figs. 2 and 3) revealed that healthy normal cerebral tissue of Tspo+/+ animals showed little retention of [18 F]PBR111 and throughout the brain low imaging signals that are close to the limit of detection (Fig. 2). In Tspo+/+ animals, this most likely reflects the constitutive, low level, mainly vascular TSPO expression in wild-type animals^28^. In contrast, Tspo−/− mice (Fig. 3) do not have any specific binding of [18 F]PBR111^11^.

After implantation of syngeneic GL261 glioblastoma cells in both wild-type Tspo+/+ and Tspo−/− mice animals, selective TSPO radioligand [18 F]PBR111 microPET/MRI/CT imaging revealed highly expressed TSPO in the tumour mass.

In wild-type Tspo+/+, the [18 F]PBR111 signal extended beyond the tumour (Fig. 2). In contrast, in TSPO−/− animals bearing the diffusely infiltrating wild-type glioma, the [18 F]PBR111 retention and signal was restricted to the tumour volume without any appreciable signal in the Tspo−/− host tissue (Fig. 3). This unprecedented contrast thus allowed detection of TSPO PET signals emanating from tumour tissue volumes smaller than 1 mm^3^. Importantly, TSPO tumour signal was already detectable at pre-symptomatic stages, i.e. in still healthy appearing animals.

### Histological tissue analysis

Microscopic neuropathological examination revealed Iba1 immunoreactive microglia/macrophages closely surrounding GL261 glioma cells in wild-type animals providing evidence that the reported intimate physical relationship between microglia/macrophages and glioblastoma cells is also reproducible in our mouse strain. GL261 cells were frequently found to be tightly “wrapped” by microglia/macrophage cell processes (Fig. 2). In Tspo−/− host brains (Fig. 3), the physical association between microglia/macrophages and implanted syngeneic GL261 glioma appeared less tight. Microglial cell processes in Tspo−/− animals were less numerous with less contact to individual GL261 tumour cells, a likely difference requiring, however separate more systematic studies.

In addition to the presence of activated microglia, astrocytes, as expected, also responded to the expanding tumour mass (Figs. 2 and 3). The GFAP astrocyte response in Tspo−/− mice appeared slightly weaker than in wild-type animals with some reactive astrocytes in Tspo−/− animals appearing dystrophic, again a subtle difference in the tissue response requiring separate investigation.

### Discussion

**Figure 4:**
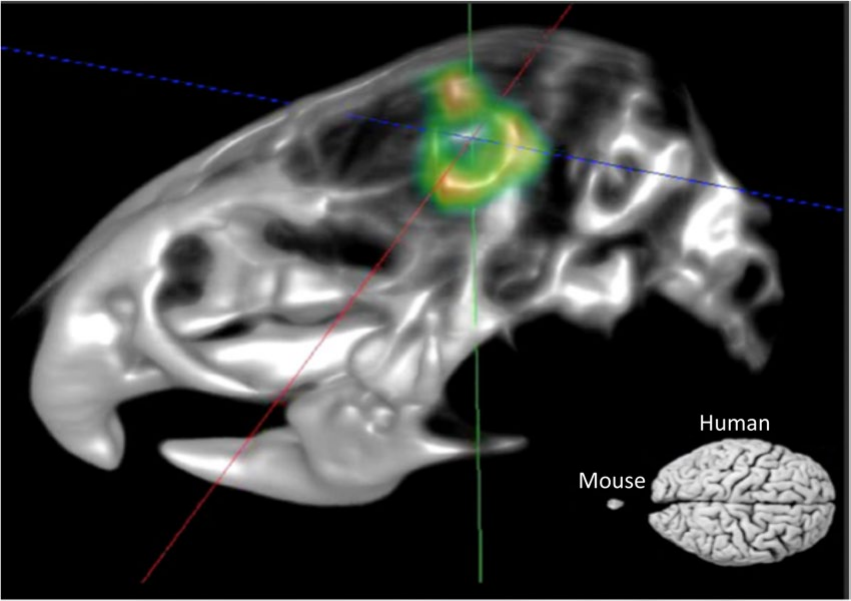
MicroPET/CT fusion image using TSPO ligand PBR111. The 3D volume-rendered image (3D visualisation option within Siemens IRW software) shows TSPO expressing tumour cells in a TSPO knock-out animal, and a size comparison of a mouse versus human brain. The image demonstrates the resolution and contrast of PET/CT imaging achievable if there is high selectivity of the radioligand and high biological contrast between the imaging target (TSPO binding-site expressing tumour) and the surrounding tissue where as a result of a complete gene knock-out (Tspo−/−) no target binding-sites can be expressed. Extra-cerebral PET signals in the lacrimal glands and in the bone, structures that commonly retain non-specifically either the ligand or free fluorine-18, have been masked. The syngeneic tumour in this null background model (global C57BL/6-Tspo^tm1GuMu(GuwiyangWurra)^-knock-out mouse) gives the unique opportunity to track tumour tissue with high contrast *in vivo* under naturalistic conditions.

Here, we introduce a model of syngeneic wild-type tumour implantation into host tissue that cannot express Tspo under any condition. Due to the global and complete absence of the Tspo gene in the Tspo−/− host tissue, it allows for the unequivocal detection and in *vivo* TSPO radioligand imaging of the infiltrating tumour with exceptional biological contrast (Fig. 4).

Our model extends recent approaches of TSPO radioligand imaging using xenografting of tumour cells into wild-type animals^29–31^.

Implantation of wild-type, i.e. Tspo gene expressing tumour in a Tspo null background has unique advantages: it enables the study of the natural tumour progression *in situ* without any confounding biological sources of Tspo expression. The reverse can also be achieved. One example, to be reported in future publications, the host organism lacking the Tspo gene can be supplied with wildtype bone marrow yielding microglia precursors (neo-microglia) that can be identified through their TSPO expression and followed in brain tissue where their interactions with glioma cells can be studied with great clarity both at the histological level and by *in vivo* imaging.

Imaging of glioma using ligands for the Tspo has been undertaken previously^32–34^ with increased Tspo protein expression strongly correlated with the grade of glioma malignancy^22,35^. The highest expression of Tspo is found in glioblastoma, especially around areas of necrosis, and a strong inverse correlation exists between Tspo expression and survival^22,35^. Recent years have seen the development of new radioligands for Tspo^23,36^, which is now a recognized target for molecular imaging and targeted drug delivery to tumours^37^. Tspo imaging has also been used successfully in an animal model of intracranial glioma to monitor therapeutic drug effects^38^.

The Tspo is functionally of particular relevance in cancer biology due to its co-regulatory role in mitochondrial energy metabolism, generation of reactive oxygen species (ROS), cell cycle regulation and apoptotic cell death^39^. The variations in the glial response between Tspo+/+ and Tspo−/− animals that the histological observations in our study seem to indicate, require further confirmation that is beyond the current report on the syngeneic xenograft model as such. It raises, however, the intriguing question as to whether Tspo-modulated local tissue responses or systemic stress regulation, which is known to involve the TSPO^40,41^, could have an influence on the progression rate (rather than incidence) of cancer^42,43^.

In summary, the dismal prognosis of patients with glioblastoma motivates the development of new treatments. The Tspo is not only a mitochondrial *in vivo* marker for molecular imaging but also a target for novel TSPO-binding drugs^44–46^, some of which have already been shown to act synergistically with other anticancer drugs, such as 5-FU^37^.
