# Author Correction: Selective, high-contrast detection of syngeneic glioblastoma *in vivo*

**DOI:** 10.1038/s41598-020-72364-1

**Published:** 2020-09-24

**Authors:** Richard B. Banati, Paul Wilcox, Ran Xu, Grace Yin, Emily Si, Eric Taeyoung Son, Mauricio Shimizu, R. M. Damian Holsinger, Arvind Parmar, David Zahra, Andrew Arthur, Ryan J. Middleton, Guo-Jun Liu, Arnaud Charil, Manuel B. Graeber

**Affiliations:** 1 Australian Nuclear Science and Technology Organization, Locked Bag 2001, Kirrawee DC, NSW 2232 Australia; 2 Medical Imaging, Faculty of Medicine and Health, The University of Sydney, 94 Mallett Street, Camperdown, NSW 2050 Australia; 3 Brain Tumour Research, Brain and Mind Centre, Faculty of Medicine and Health, The University of Sydney, 94 Mallett Street, Camperdown, NSW 2050 Australia; 4 Molecular Neuroscience, Brain and Mind Centre, Faculty of Medicine and Health, The University of Sydney, 94 Mallett Street, Camperdown, NSW 2050 Australia

Correction to: *Scientific Reports* https://doi.org/10.1038/s41598-020-67036-z, published online 19 June 2020

This Article contains errors.

In the Materials and Methods section, under the subheading ‘PET/CT and MR imaging’,

“Mice, anaesthetized (5% (v/v) isoflurane and maintained at 1–2%), were scanned using a small-animal Inveon PET/CT scanner (Siemens, Knoxville, TN) following methods described previously^26,27^.”

should read:

“The TSPO selective radioligand [18F] PBR111 was produced at the ANSTO/University node of the Sydney National Imaging Facility following accredited good manufacturing practice(GMP). Mice, anaesthetized (5% (v/v) isoflurane and maintained at 1–2%), were scanned using a small-animal Inveon PET/CT scanner (Siemens, Knoxville, TN) following methods described previously^26,27^.”

In the Acknowledgements,

“This work was partially funded by the Australian Institute of Nuclear Science and Engineering (AINSE), Research Project ALNGRA14524, and Cure for Life Foundation/Cure Brain Cancer Australia.”

should read:

“This work was carried out at the National Imaging Facility (joined node ANSTO/University of Sydney and the University of New South Wales) and partially funded by the Australian Institute of Nuclear Science and Engineering (AINSE), Research Project ALNGRA14524, and Cure for Life Foundation/Cure Brain Cancer Australia.”

